# Supplementary material for: Common and cell-type specific responses to anti-cancer drugs revealed by high throughput transcript profiling
Source: Nat Commun. 2017 Oct 30;8:1186. doi: 10.1038/s41467-017-01383-w (PMC5662764; doi:10.1038/s41467-017-01383-w)
Supplement: Supplementary file 1 — Supplementary Information [file 41467_2017_1383_MOESM1_ESM.pdf]

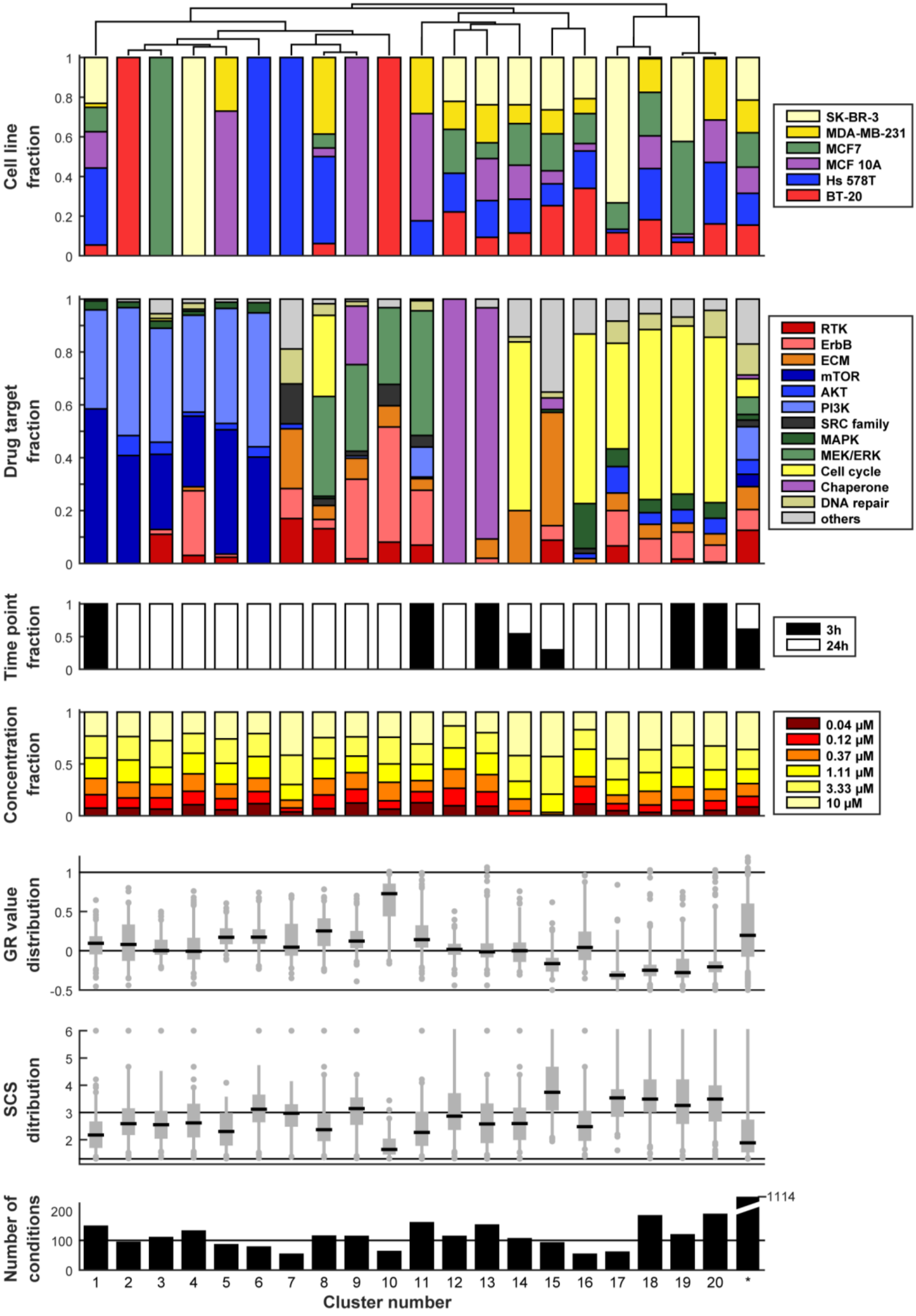
Cell line fraction
SK-BR-3
MDA-MB-231
MCF7
MCF 10A
Hs 578T
BT-20
Drug target fraction
RTK
ErbB
ECM
mTOR
AKT
PI3K
SRC family
MAPK
MEK/ERK
Cell cycle
Chaperone
DNA repair
others
Time point fraction
3h
24h
Concentration fraction
0.04 μM
0.12 μM
0.37 μM
1.11 μM
3.33 μM
10 μM
GR value distribution
SCS ditribution
Number of conditions
1114
Cluster number

Supplementary Figure 1:

In each panel, columns correspond to a cluster of significant perturbations (SCS>1.3), ordered as in Fig. 1a. The clusters are grouped based on the cosine distance between their centroids (dendrogram at the top). The last column regroups the perturbations that are not systematically part of a specific cluster (see methods). The top panel shows fraction of each cell line in the clusters, the second the fraction of drug targets, the third fraction of time points, the fourth the fraction of doses. The fifth and sixth panels show the box plots of the GR and SCS values, corresponding the perturbations in the cluster. The last panel shows the number of perturbations in each cluster. The group of perturbations that does not reliably group with any of the 20 identified clusters is labelled with a star and is comprised of 1114 perturbations.

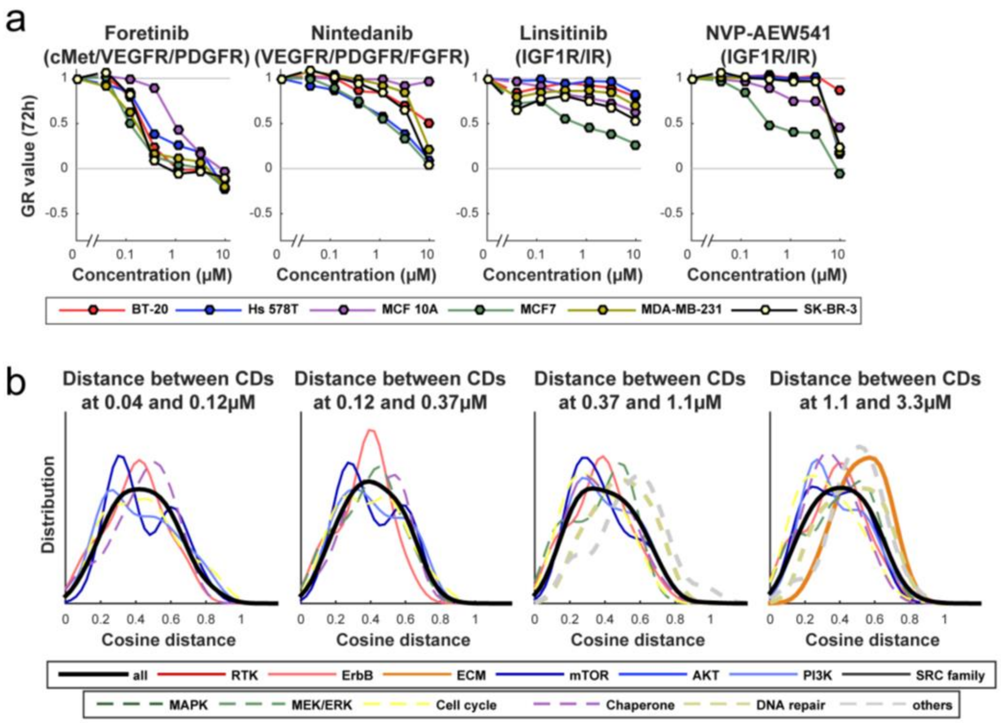

Supplementary Figure 2:

(a) Dose response curves across six cell lines for a subset of drugs that exhibit significant responses at the level of L1000 signature and phenotype. A GR value of one represents growth at the same rate as the untreated control, a value of zero denotes a complete cytostatic response, and a negative value denotes a cytotoxic response. (b) Distribution of the pairwise cosine distance between significant perturbations for the same drug and cell line where only the concentration is changed as indicated in the plot. Each curve is a smooth density plot by class of drug target. Each distribution is based on an average of 44, but at least 20, different conditions that generally comprises all cell lines. The black line shows the distribution of all perturbations and colored lines show the distribution for each class of drug targets.

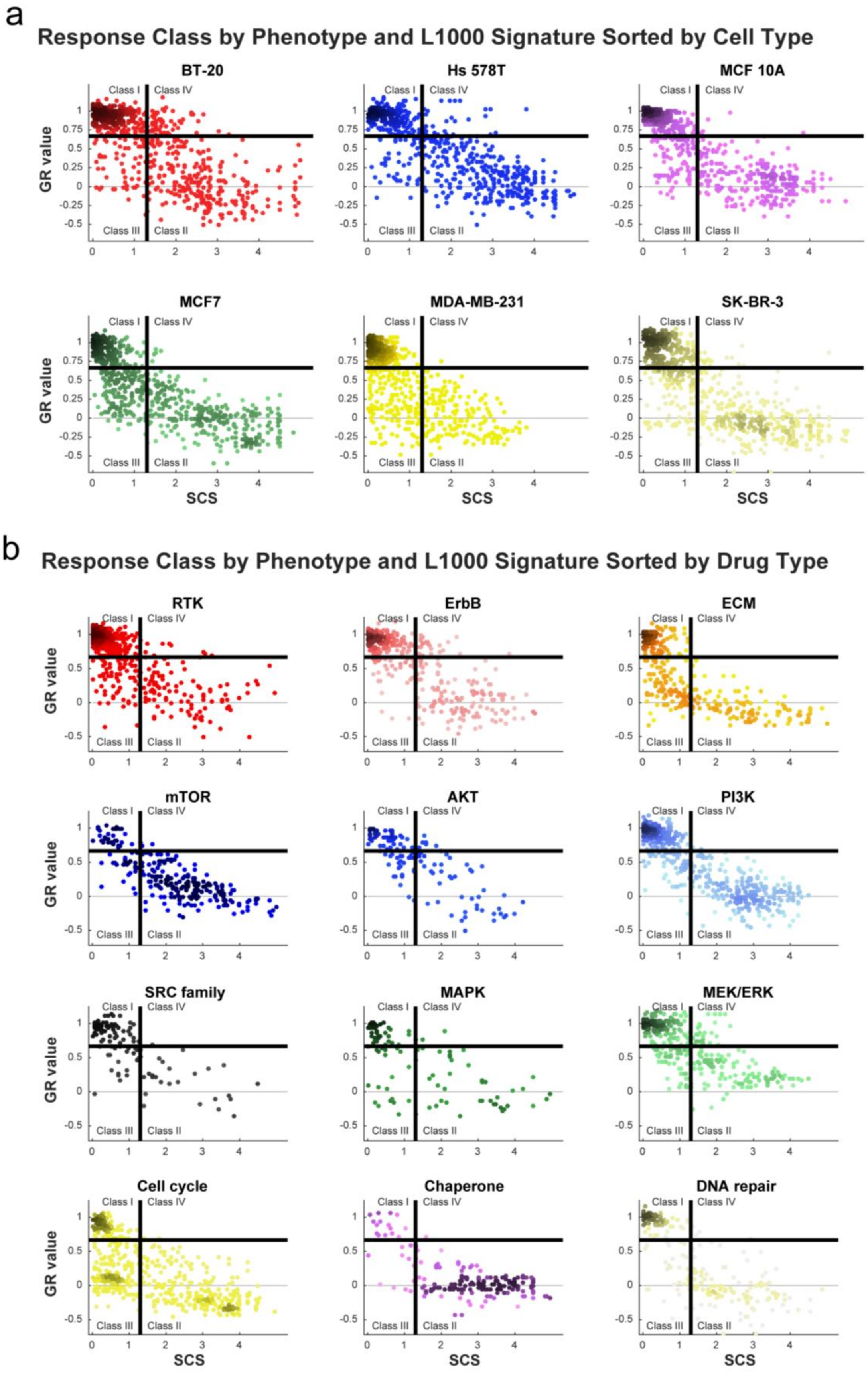
a
Response Class by Phenotype and L1000 Signature Sorted by Cell Type
BT-20
Hs 578T
MCF 10A
MCF7
MDA-MB-231
SK-BR-3
Class I
Class II
Class III
Class IV
GR value
SCS
b
Response Class by Phenotype and L1000 Signature Sorted by Drug Type
RTK
ErbB
ECM
mTOR
AKT
PI3K
SRC family
MAPK
MEK/ERK
Cell cycle
Chaperone
DNA repair

Supplementary Figure 3:

Scatterplot showing the phenotypic response (GR_{AOC}) and molecular response (SCS of the L1000 signature) for all perturbations, separated into individual plots for each drug class. Classes based on a cutoff of $GR_{AOC=}$0.66 and SCS=1.3. Class I: Non-responsive by both measures; Class II: responsive by both measures; Class III: responsive by GR_{AOC} and not by SCS; Class IV: responsive by SCS and not GR_{AOC}.

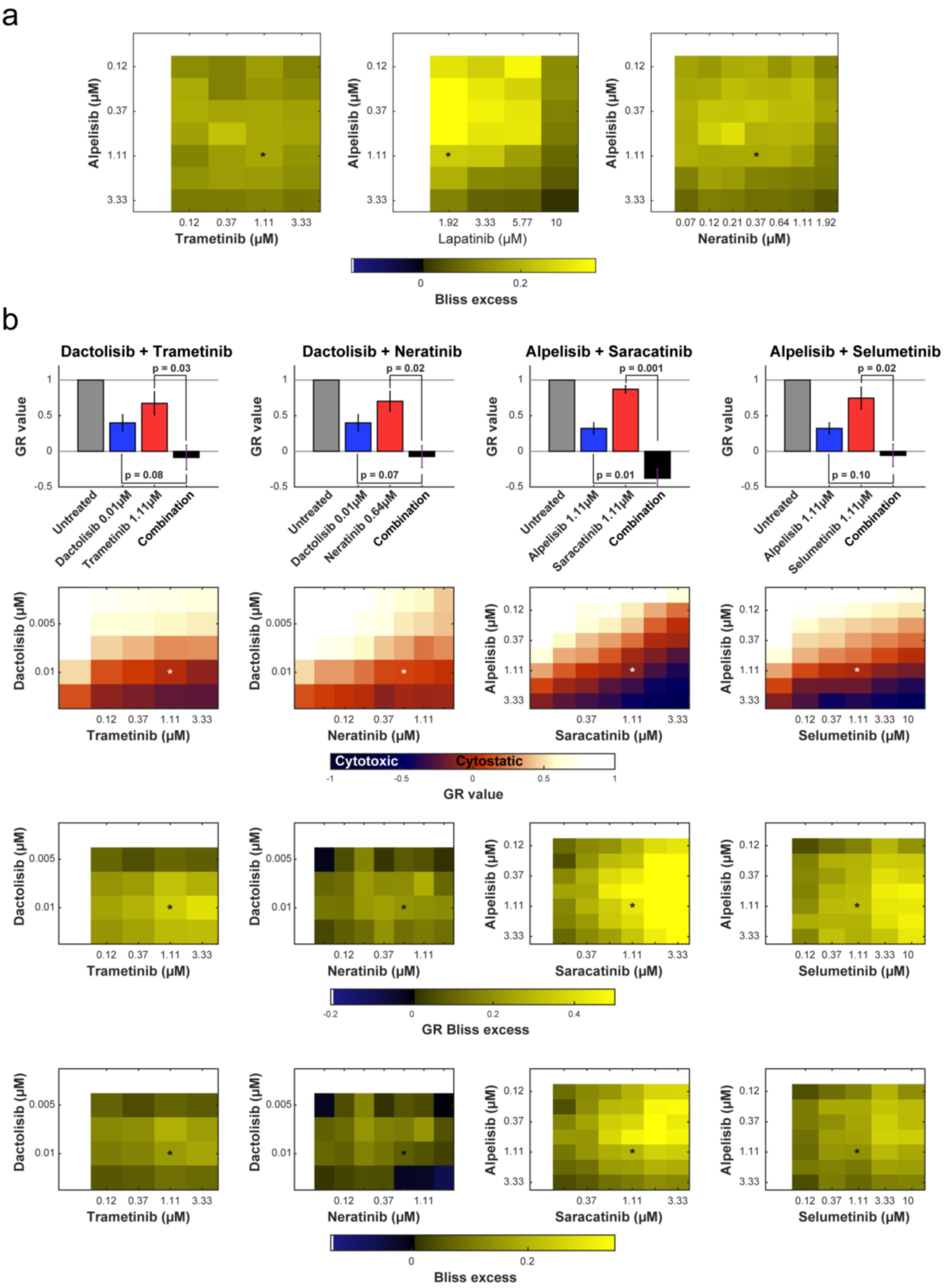
a
Alpelisib (μM)
0.12
0.37
1.11
3.33
Trametinib (μM)
0.12 0.37 1.11 3.33
Lapatinib (μM)
1.92 3.33 5.77 10
Neratinib (μM)
0.07 0.12 0.21 0.37 0.64 1.11 1.92
0
0.2
Bliss excess
b
Dactolisib + Trametinib
Dactolisib + Neratinib
Alpelisib + Saracatinib
Alpelisib + Selumetinib
GR value
p = 0.03
p = 0.08
p = 0.02
p = 0.07
p = 0.001
p = 0.01
p = 0.02
p = 0.10
Untreated
Dactolisib 0.01μM
Trametinib 1.11μM
Neratinib 0.64μM
Alpelisib 1.11μM
Saracatinib 1.11μM
Selumetinib 1.11μM
Combination
Dactolisib (μM)
0.005
0.01
Saracatinib (μM)
Selumetinib (μM)
0.12 0.37 1.11 3.33 10
Cytotoxic
Cytostatic
-1
-0.5
0
0.5
1
GR value
-0.2
0
0.2
0.4
GR Bliss excess
0
0.2
Bliss excess

Supplementary Figure 4:

(a) Excess over Bliss score for the exposure of BT-20 cells to combinations of alpelisib with trametinib, lapatinib, or neratinib. (b) Exposure of BT-20 cells to combinations of dactolisib with trametinib or neratinib and alpelisib with saracatinib or selumetinib. Shown is the phenotypic effect of the combination of individual drug doses and heatmaps indicating the phenotypic response, the GR_{EOB}, and the EOB for a range of concentrations of all drug combinations. Histograms show the mean of three biological repeats and error bars indicated the standard error of the mean; p-value is based on a t-test. Heatmaps show data from one out of three biological replicates.

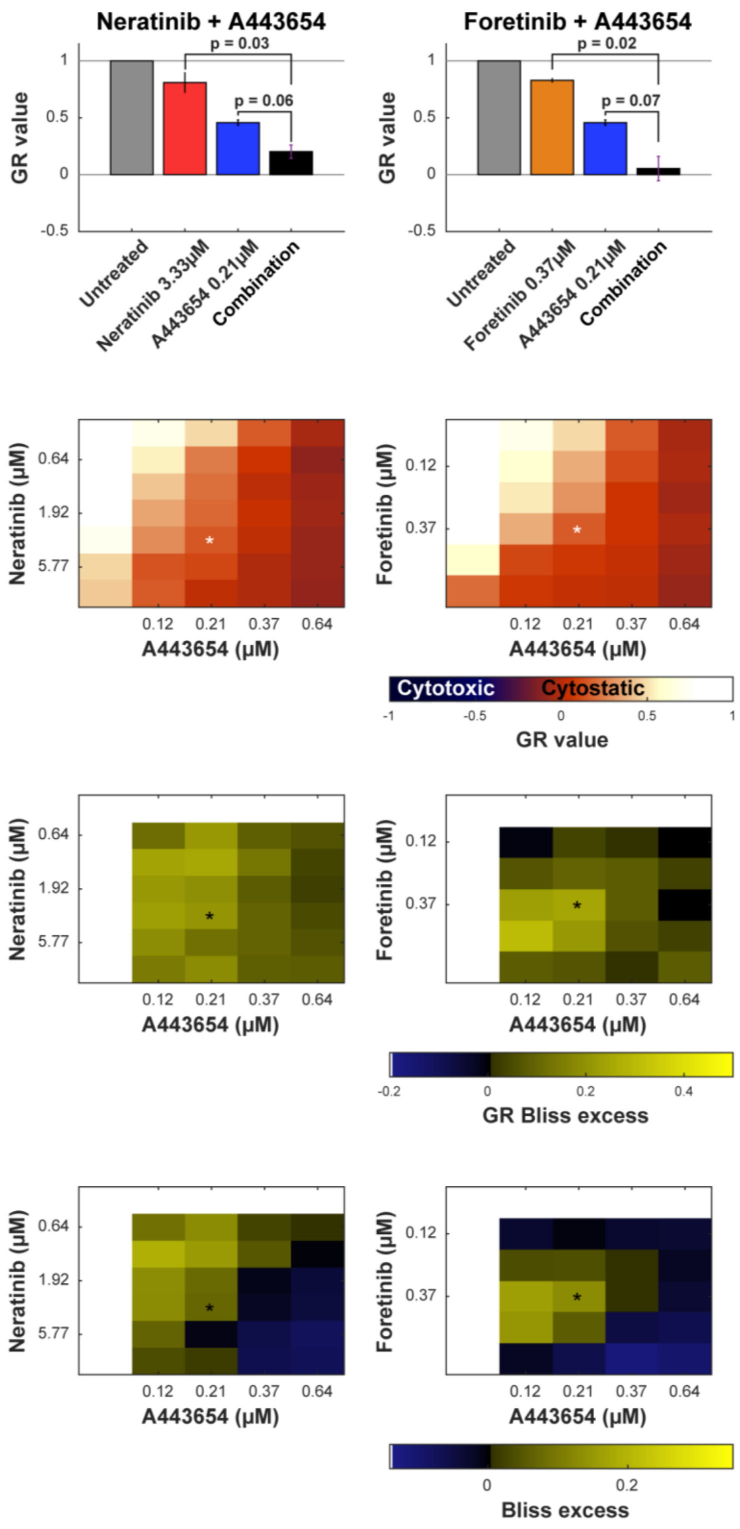

Neratinib + A443654
Foretinib + A443654
Neratinib + Foretinib
GR value
p = 0.03
p = 0.06
p = 0.02
p = 0.07
p = 0.09
p = 0.14
Untreated
Neratinib 3.33μM
A443654 0.21μM
Foretinib 0.37μM
Combination
Neratinib (μM)
Foretinib (μM)
A443654 (μM)
Cytotoxic
Cytostatic
GR value
GR Bliss excess
Bliss excess

Supplementary Figure 5:

Exposure of Hs 578T cells to pairwise combinations of neratinib, foretinib and A443654. Shown is the phenotypic effect of the combination of individual drug doses and heatmaps indicating the phenotypic response, the GR_{EOB}, and the EOB for a range of concentrations of all drug combinations. Histograms show the mean of three biological repeats and error bars indicated the standard error of the mean; p-value is based on a t-test. Heatmaps show data from one out of two biological replicates.

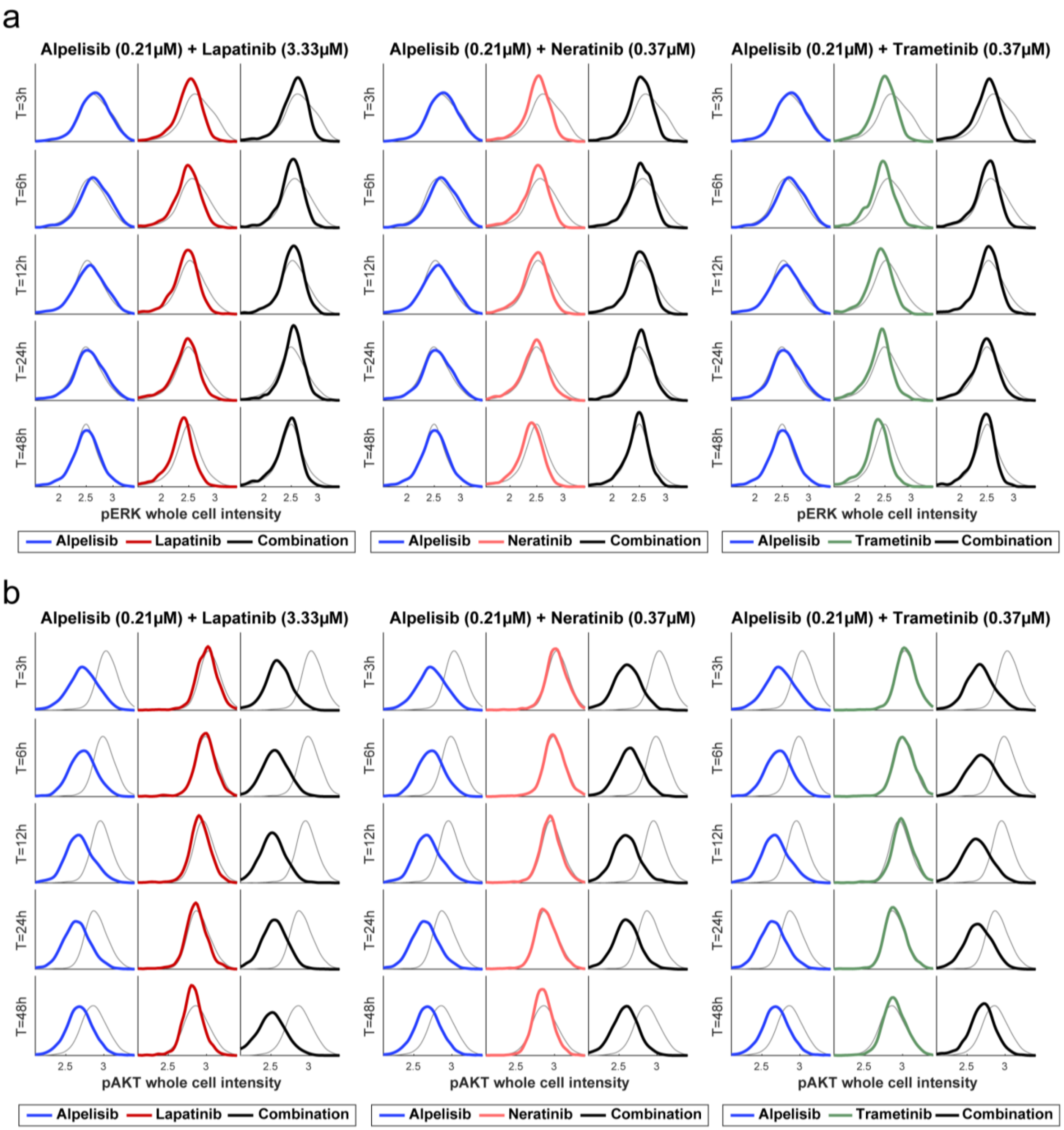

Supplementary Figure 6:

Distribution of the (a) pERK and (b) pAKT levels measured by quantitative immunofluorescence microscopy at the indicated timepoints for BT-20 cells treated with single drugs, in combination, and for untreated control cells. Combinations of alpelisib with lapatinib (left), neratinib (middle), and trametinib

(right). Gray lines show untreated controls, blue alpelisib, red the EGFR or MEK inhibitor treatments, and black the combination treatments. Data shown is one out of three biological replicates.

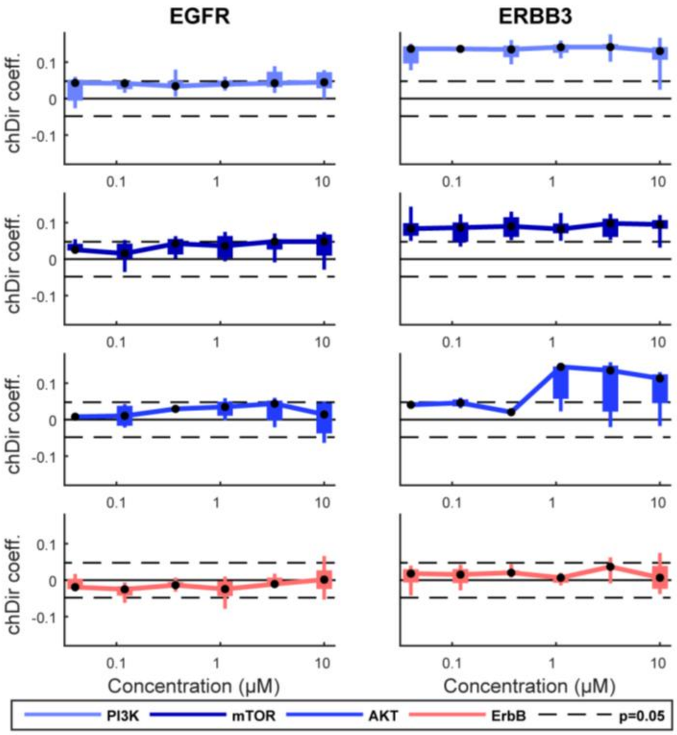

Supplementary Figure 7:

Boxplot of the EGFR (left) and ERBB3 (right) levels of the coefficients in the characteristic direction aggregated by drug targets (PI3K, mTOR, AKT, and ErbB) shown for each concentration. Black lines represent the median, boxes the interquartile range, and lines the 5% and 95% quantiles.

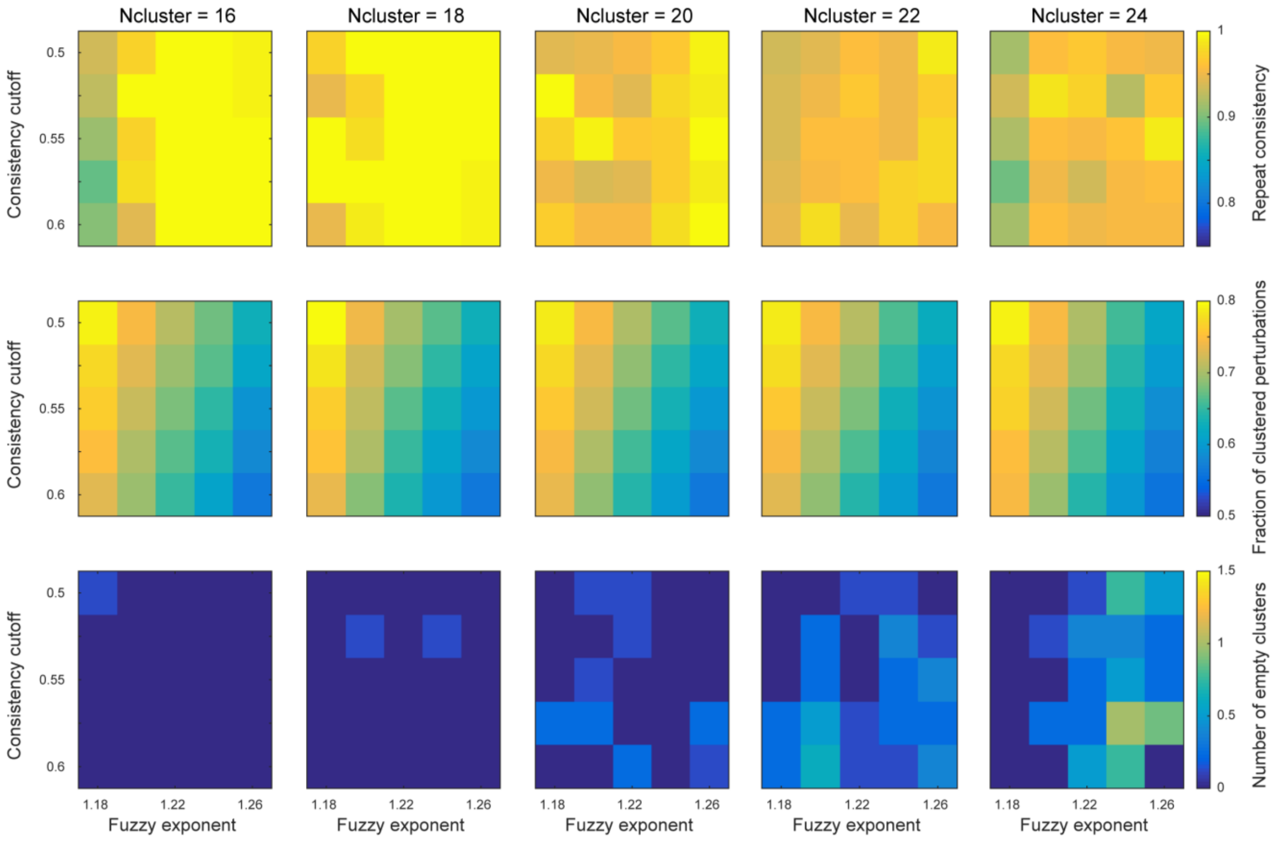

Supplementary Figure 8:

Benchmarking fuzzy clustering: average results for eight independent clustering runs with different parameters. Top row shows the consistency of the clustering across multiple repeats (with fraction of perturbations in matching clusters shown). Middle row shows the fraction of perturbations in a cluster. Bottom row shows the number of empty clusters. For each plot, x-axis is the fuzzy exponent; y-axis is the cutoff for accepting perturbations based on clustering consistency. Each column of plots shows a different number of clusters.
