# Supplementary material for: Common and cell-type specific responses to anti-cancer drugs revealed by high throughput transcript profiling
Source: Nat Commun. 2017 Oct 30;8:1186. doi: 10.1038/s41467-017-01383-w (PMC5662764; doi:10.1038/s41467-017-01383-w)
Supplement: Supplementary file 3 — Description of Additional Supplementary Files [file 41467_2017_1383_MOESM3_ESM.pdf]

Description of Additional Supplementary Files

File Name: Supplementary Data 1
Description: List of all drugs with their assigned nominal targets and the target class as used in this paper.

File Name: Supplementary Data 2
Description: List of all growth rate values and relative cell counts corresponding to all conditions measured by the L1000 transcription assay.

File Name: Supplementary Data 3
Description: All drug perturbations at 3 hours listed by their response class, based on the Signature Consistency Score and GR value.

File Name: Supplementary Data 4
Description: All drug perturbations at 24 hours listed by their response class, based on the Signature Consistency Score and GR value.

File Name: Supplementary Data 5
Description: Values of protein abundance measured by shotgun proteomics.

File Name: Supplementary Data 6
Description: Values of phospho-peptide abundance measured by shotgun proteomics.

File Name: Supplementary Data 7
Description: List of proteins in the RTK pathway for the mass spectrometry analysis.

File Name: Supplementary Data 8
Description: List of proteins in the PI3K/AKT pathway for the mass spectrometry analysis.

File Name: Supplementary Data 9
Description: List of proteins in the MAPK pathway for the mass spectrometry analysis.
